# Maternal and neonatal outcomes in women with COVID-19: A retrospective cohort study

**DOI:** 10.18502/ijrm.v22i10.17667

**Published:** 2024-12-02

**Authors:** Malihe Nourollahpour Shiadeh, Ommolbanin Zare, Mahmood Moosazadeh, Azadeh Kiapour, Sima Elyasi

**Affiliations:** ^1^Sexual and Reproductive Health Research Centre, Mazandaran University of Medical Sciences, Sari, Iran.; ^2^Department of Midwifery, Comprehensive Health Research Center, Babol Branch, Islamic Azad University, Babol, Iran.; ^3^Gastrointestinal Cancer Research Center, Non-communicable Disease Institute, Mazandaran University of Medical Sciences, Sari, Iran.; ^4^Department of Statistics, Babol Branch, Islamic Azad University, Babol, Iran.; ^5^Department of Nursing and Midwifery, Mazandaran University of Medical Sciences, Sari, Iran.

**Keywords:** Pregnancy, COVID-19, Outcome.

## Abstract

**Background:**

Considering the conflicting evidence that exists regarding the effect of the coronavirus disease 2019 (COVID-19) on pregnancy and newborn outcomes, it is necessary to conduct this research during the first year in the north of Iran, a highly visited and trafficked place, which increases the possibility of contracting the virus and severity of the infection.

**Objective:**

This study aimed to compare maternal and neonatal outcomes in pregnant women with and without COVID-19.

**Materials and Methods:**

In this retrospective cohort study, data from 160 pregnant women referred to 3 hospitals in Mazandaran, Iran from April 2020 to March 2021 were extracted from their medical records using checklists. Participants were divided into 2 groups: group A) 80 pregnant women with COVID-19 and group B) 80 noninfected pregnant women. All information related to maternal and neonatal outcomes were extracted and compared from the files of the 2 groups. The results obtained from comparing 2 groups of pregnant women from the result of the reverse-transcription polymerase chain reaction test were analyzed.

**Results:**

The most common symptoms in the COVID-19 group were fever and cough. Pregnancy complications such as abortion, pre-eclampsia, and premature birth were not significant between groups (p = 0.4, 0.45, 0.45, respectively). No statistically significant difference was observed between groups regarding maternal and neonatal outcomes.

**Conclusion:**

Despite the peak and severity of the COVID-19 disease in the first year of the pandemic, it seems that it had no effect on maternal and neonatal outcomes in each trimester of pregnancy in affected women.

## 1. Introduction

The coronavirus disease (COVID-19) caused by acute respiratory syndrome coronavirus (SARS-CoV-2) has become a global health emergency ever since it was declared a pandemic on March 11, 2020, by World Health Organization. SARS-CoV-2 can lead to severe illness in older people with chronic diseases; however, anyone can contract the disease, from adults, adolescents, and children to pregnant women and infants (1).

A special immunological adaptation is developed in women during pregnancy, which is necessary for the tolerance of fetal allograft. This transient immunosuppressed state is modulated by suppression of t-cell activity, thereby predisposing pregnant women to viral diseases (2).

Pregnancy and fetal consequences of COVID-19 are still not fully known. In addition, the potential for vertical transmission of COVID-19 is currently unclear (3). In some studies, pregnancy outcomes such as premature delivery rate of 17%, cesarean section rate of 65% (4), as well as fetal outcomes of COVID-19 such as prematurity (39–47%), intrauterine growth restriction (10%), and abortion (2%) have been reported. On the other hand, while pregnant women with COVID-19 have been considered a high-risk group, the effect of this disease on pregnancy is still unclear. In some studies, it was reported that the infection with SARS-COV-2 is not related to the increase in the rate of premature birth and that the increase in the rate of cesarean section was due to iatrogenic factors (5).

Also, there is limited evidence of intrauterine vertical transmission of COVID-19 in pregnant women afflicted with this disease (6). However, in some studies, transmission of COVID-19 through the placenta has been reported (5). In other investigations, there has been no strong evidence of vertical transmission of COVID-19 in the third trimester of pregnancy (7). However, the risk of vertical transmission is not clear when COVID-19 occurs in the first or second trimesters, or when there is a long interval from clinical manifestations to delivery (8).

However, there is conflicting evidence regarding the impact of COVID-19 on pregnancy outcomes. Moreover, there is not enough evidence to conclude any definite harmful effects of COVID-19 during pregnancy (6). In addition, most studies have been conducted in countries with a favorable socio-economic status as well as access to good healthcare services (9).

Considering the conflicting evidence that exists regarding the effect of the COVID-19 on pregnancy and newborn outcomes, it is necessary to conduct this research during the first year in the north of Iran, which is a highly visited and trafficked place, increasing the possibility of contracting the coronavirus and the severity of the infection. Therefore, more research is needed on pregnant women with COVID-19 around the world. The present study was conducted on pregnant women with a larger sample size, and by sampling from several treatment centers in the first year of the COVID-19 epidemic. This study aimed to investigate and compare maternal and neonatal outcomes in pregnant women with and without COVID-19.

## 2. Materials and Methods

In this retrospective cohort study, data of 160 pregnant women who referred to 3 hospitals affiliated with Mazandaran University of Medical Sciences, Mazandaran, Iran (Imam Ali hospital in Amol, Imam Khomeini hospital in Sari, and Shahid Rajaei hospital in Tonekabon) from April 2020 to March 2021, were extracted from participants' medical files.

Participants were divided into 2 groups (group A: 80 pregnant women with COVID-19, group B 80 noninfected pregnant women).

All information related to maternal outcomes including abortion, stillbirth, bleeding in the third trimester, postpartum hemorrhage, gestational diabetes, pre-eclampsia, oligohydramnios, meconium staining, preterm labor, preterm labor, fetal distress, asphyxiation, type of delivery and neonatal outcomes including gestational age at birth, birth weight, Apgar in 1 and 5 min, low birth weight, admission to the neonatal ward and neonatal intensive care unit were extracted and compared from the files of the 2 groups.

The sampling method in this study was as follows: Mazandaran Province was initially classified into 3 parts: east, west, and central, and then several cities in Mazandaran province such as Amol, Sari, and Tonekabon were targeted from this category based on the statistics of COVID-19 incidence.

In the next stage, Imam Khomeini, Imam Ali, and Shahid Rajaei hospitals were selected until the sample size of each city's quota was reached.

In the last stage, the required number of files of infected pregnant women and the same number of noninfected pregnant women were chosen using nonprobability available sampling. In addition to files from the health center, hospital files were also used to collect more accurate and complete information.

2 groups were controlled with a limiting approach and through the application of entry and exit criteria in terms of possible confounding variables.

In this part of the study, we completed checklists related to demographic information, fertility, COVID-19, and newborns by referring to the mentioned hospitals and using the medical files of participants.

Inclusion criteria were as follows: living in Mazandaran province, aged between 18 and 45 yr and being pregnant, infection with COVID-19 confirmed by reverse-transcription polymerase chain reaction test in group A, and a negative reverse-transcription polymerase chain reaction test in group B during hospitalization.

Exclusion criteria were having multiple pregnancies, and lack of enough data in the medical record.

### Sample size

Based on a literature review as well as maternal and fetal outcomes (10), and by using the Pocock formula (α = 0.05; β = 0.2; fetal distress in group A: P1 = 0.05; fetal distress in group B: P2 = 0.2) the minimum sample number in each group was estimated to be 72 individuals with a 10% chance of dropping out, 80 women were selected for each group. 


$$n=\frac{{\left(Z_{1-\alpha /2}+Z_{1-\beta }\right)}^{2}\left[P_{1}\left(1-P_{1}\right)+P_{2}\left(1-P_{2}\right)\right]}{({p_{1}-p_{2})}^{2}}$$

### Ethical Considerations

This study was approved by the Ethics Committee of Mazandaran University of Medical Sciences, Mazandaran, Iran (Code: IR.MAZUMS.REC.1401.315).

### Statistical Analysis

In this research, descriptive and analytical statistics were employed for analysis using Statistical Package for the Social Sciences, version 21.0, SPSS Inc., Chicago, Illinois, USA (SPSS) SPSS21. Descriptive statistics included frequency distribution tables and mean and standard deviation indices to describe the characteristics of subjects, and to evaluate the information in the checklists.

The normality of the distributions was tested using the Kolmogorov-Smirnov test. To check the qualitative variables, Chi-square, and Fisher's exact test were used to check and compare the quantitative variables with normal distribution, independent *t* test was utilized in this research. A significance level of p < 0.05 was considered.

## 3. Results

Of the 160 pregnant women included in this study, 80 women with COVID-19 were informed in group A and 80 normal pregnant women were assigned to group B. 55 women from group A and 58 women from group B did not have chronic diseases. The demographic characteristics and medical history of participants are presented in table I.

The groups were similar in terms of maternal age, body mass index, number of pregnancies, gestational age, assisted reproductive technology, history of abortion, cesarean section, and preterm delivery (p > 0.05).

In the COVID-19 group, the most common symptoms were fever (25%) and cough (23.8%), and the least frequent symptoms were shortness of breath (11.3%), fatigue (8.8%), body pain (7.5%), headache (6.3%), diarrhea (5%), sore throat (3.8%), lack of sense of smell (2.5%), taste (1.3%), and asymptomatic (5%). A total of 43, 23, and 10 individuals were treated with antibiotics, viral therapy, and hydroxychloroquine or chloroquine, respectively.

Out of 80 pregnant women with COVID-19, 58 were hospitalized in the obstetrics department, 22 were hospitalized in the intensive care unit, and 4 needed mechanical ventilation. The length of hospitalization of individuals was 2–12 days. 32 individuals had a CT report based on typical lesions related to COVID-19, of whom 19 women had radiological findings of ≤ 25% and 13 had radiological findings of 25–50%.

Pregnancy complications were compared in pregnant women with and without COVID-19 (Table II). Pregnancy complications such as abortion, stillbirth, third-trimester bleeding, postpartum hemorrhage, gestational diabetes, pre-eclampsia, oligohydramnios, amniotic fluid stained with meconium, premature labor, and premature rupture before labor were not significant between the 2 groups.

Pregnancy and newborn outcomes are presented in table III. No statistically significant difference was observed between the 2 groups in the rate of vaginal delivery and cesarean section. One individual had a cesarean delivery due to COVID-19. No significant difference was observed in the condition of infants in the 2 groups. The mean age at birth was not significantly different between the 2 groups (p = 0.35). No significant differences were observed between the 2 groups in terms of fetal distress (p = 0.3), neonatal respiratory distress (p = 0.58), hospitalization in the neonatal unit (p = 0.38), and transfer to neonatal intensive care unit (p = 0.5). There was one infant with COVID-19 in the group of women afflicted with this disease.

**Table 1:** The demographic characteristics and medical history of participants

**Variables**		**Group A **	**Group B **	**P-value**
Age (yr)*		29.5 ± 6.1 (30, 9)	30.1 ± 5.7 (30, 8)	0.47
BMI (kg/m^2^)*		25.86 ± 4.47 (25, 7)	26.8 ± 4.02 (26, 4.75)	0.18
Gravida**				
	**Primiparity**	34 (42.5)	33 (41.25)	
	**Multiparity**	46 (57.5)	47 (58.75)	0.86
Gestational age (wk)*		37.83 ± 2.95 (38, 1)	37.46 ± 3.85 (38, 1)		0.5
Previous abortion***		13 (16.25)	13 (16.25)	1
ART***		0	1 (1.25)	0.3
Previous cesarean section**		22 (27.5)	26 (32.5)	0.1
Previous preterm labor***		2 (2.5)	2 (2.5)	1
Chronic disease***		25 (31.25)	23 (28.75)	0.31
Diabetes***		3 (3.75)	1 (1.25)	0.62
Chronic hypertension***		3 (3.75)	2 (2.5)	0.5
Hypothyroidism***		17 (21.25)	16 (20)	0.81
Asthma***		2 (2.5)	3 (3.75)	0.98
Thalassemia minor***		0	1 (1.25)	0.5
*Data presented as Mean ± SD, median (interquartile range), independent *t* test. **Data presented as n (%), Chi-square. ***Data presented as n (%), Fisher's exact test. BMI: Body mass index, ART: Assisted reproduction treatment				

**Table 2:** Pregnancy complications between groups

**Variables**	**Group A **	**Group B **	**P-value**
**Abortion**	1 (1.25)	1 (1.25)	0.6
**Stillbirth**	2 (2.5)	1 (1.25)	0.5
**Bleeding in the third trimester**	2 (2.5)	0	0.5
**PPH**	1 (1.25)	4 (5)	0.4
**Gestational diabetes**	5 (6.25)	5 (6.25)	0.6
**Pre-eclampsia**	5 (6.25)	3 (3.75)	0.4
**Oligohydramnios**	0	2 (2.5)	0.5
**Meconium staining **	8 (10)	4 (5)	0.4
**Preterm labor**	5 (6.25)	8 (10)	0.6
**PROM**	1 (1.25)	0	0.5
**Fetal distress**	10 (12.5)	8 (10)	0.4
**Asphyxiation**	13 (16.25)	10 (12.5)	0.6
Data presented as n (%), Fisher's exact test. PPH: Postpartum hemorrhage, PROM: Premature rupture of membranes			

**Table 3:** Pregnancy-neonatal outcomes between groups

**Variables**		**Group A **	**Group B **	**P-value**
Gestational age at birth (wk)*		37.13 ± 3.3 (38, 1)	36.6 ± 4.2 (38, 1)	0.35
Spontaneous delivery**		41 (51.25)	43 (53.75)	0.6
Cesarean section**		39 (48.75)	37 (46.25)	0.3
	**Repeat**	24 (30)	25 (31.25)	0.23
	Dystocia***	6 (7.5)	3 (3.75)	0.3
	COVID-19***	1 (1.25)	0	0.4
	Fetal distress***	8 (10)	10 (12.5)	0.3
Birth weight (gr)*		2896 ± 590.95 (2965, 645)	2937 ± 888.87 (3070, 860)	0.73
**Apgar score**				
	**1 min**	8.5 ± 1.9 (9, 0)	8.6 ± 1.6 (9, 0)	0.56
	**5 min**	9.5 ± 1.96 (10, 0)	9.6 ± 1.8 (10, 0)	0.77
LBW***		17 (21.25)	14 (17.5)	0.3
Admission to the neonatal ward***		5 (6.25)	10 (12.5)	0.38
Admission to the NICU***		6 (7.5)	6 (7.5)	0.5
*Data presented as Mean ± SD (interquatile range), independent *t* test. **Data presented as n (%), Chi-square. ***Data presented as n (%), Fisher's exact test. LBW: Low birth weight, NICU: Neonatal intensive care unit				

## 4. Discussion

In this study, the most common symptoms related to COVID-19 were fever (20%) and cough (19%). Less common symptoms included shortness of breath, fatigue, body pain, headache, diarrhea, and sore throat. Chen et al., reported that the most common symptoms of COVID-19 in 9 pregnant women were fever and cough (11). In another study, pregnant women reported symptoms of fever, cough, and shortness of breath (12, 13). In the current research, treatment options for pregnant women with COVID-19 were antibiotics, antiviral treatment, and hydroxychloroquine, which is similar to other studies (10, 14).

In the present research, out of 80 pregnant women with COVID-19, 58 were hospitalized in the obstetrics department, 22 were hospitalized in the intensive care unit, and 4 required mechanical ventilation. According to other investigations, pregnant women are admitted to the intensive care unit more often than nonpregnant women. However, this may be due to the low threshold for intensive care unit admission in pregnant women afflicted with COVID-19 (12). There are also case reports from countries such as the United States of America indicating that women need invasive mechanical ventilation (15).

Based on the findings of this research, a statistically significant difference was observed between the 2 groups in terms of pregnancy complications such as pre-eclampsia, gestational diabetes, oligohydramnios, premature rupture of fetal membranes, amniotic fluid stained with meconium, premature labor, abortion, stillbirth, and postpartum hemorrhage. The results of other studies also indicated no increase in perinatal complications of women with COVID-19, including severe pre-eclampsia, premature rupture of fetal membranes, amniotic fluid stained with meconium, premature delivery, fetal distress, asphyxia, and postpartum hemorrhage (10, 12, 16).

In one study, it was reported that the rate of pregnancy complications did not increase in the group of individuals afflicted with COVID-19. Even when the total cases of pre-eclampsia, placental abruption, and stillbirth were compared between the previous year and during the pandemic, no significant difference was distinguished (9). Another research reported no significant change in the frequency of other adverse pregnancy outcomes such as stillbirth during the COVID-19 pandemic (17).

In the present study, pregnancy and newborn outcomes were similar to those of noninfected pregnant women, including the type of delivery, birth weight of infants, Apgar score, and admission of the infant to the neonatal unit and intensive care unit. These findings were in line with studies conducted in other provinces of Iran (10, 18) as well as investigations conducted in other countries (9, 12). However, in other studies, the cesarean section rate is higher in women with confirmed (COVID-19) (19, 20). This may be related to the period in which these studies were conducted, particularly if data collection occurred at the peak of or early in the epidemic, when obstetricians may have selected a lower threshold for cesarean section (12).

In a study comparing neonatal outcomes between pregnant women with and without COVID-19, no difference was reported in the 5-min Apgar score ( < 7) (21) indicating that COVID-19 does not increase respiratory distress in infants (12). In another research, neonatal outcomes were not significantly different between infected and noninfected groups, and 5.9% of infants from the COVID-19 group were transferred to the intensive care unit. It seems that the admission rate in the neonatal department was low due to COVID-19 in babies born to mothers with this disease, as such babies are mildly affected by this disease (12). Also, in the present study, there was no infant with COVID-19 in the group of women with COVID-19, which is also consistent with other investigations (10, 22).

Our research has several strengths, including a larger sample size and the inclusion of a control group for comparison, as well as being a multicenter, retrospective cohort study that could rule out selection bias. This study also has several limitations as follows. Due to the retrospective nature of the research, there were no tests on samples of placenta, amniotic fluid, umbilical cord blood, and vaginal mucus, so the conclusion about the lack of vertical transmission potential is weak. It is suggested that further studies should be conducted in other parts of Iran with a larger number of samples from participants with chronic disease to investigate maternal, pregnancy, and neonatal outcomes.

## 5. Conclusion

Despite the peak and severity of the COVID-19 disease in the first year of the pandemic, it seems that it had no effect on maternal and neonatal outcomes in each trimester of pregnancy in affected women. However, it is suggested that more studies be conducted in other parts of Iran with a larger number of participants with chronic disease to investigate maternal and neonatal outcomes.

##  Data Availability

Data supporting the findings of this study are available upon reasonable request from the corresponding author.

##  Author Contributions

M. Nourollahpour Shiadeh and O. Zare: Concept and design. O. Zare: Had full access to all of the data in the study and takes responsibility for the integrity of the data and the accuracy of the data analysis, Supervision. M. Moosazadeh: Acquisition, analysis, or interpretation of data. Critical revision of the manuscript for important intellectual content: All authors. A. Kiapour: Statistical analysis. S. Elyasi: Drafting of the manuscript.

##  Conflict of Interest

The authors declare that there is no conflict of interest.
